# The relationship between theory of mind and executive functions in major depressive disorders: A review

**DOI:** 10.3389/fpsyt.2022.980392

**Published:** 2022-08-16

**Authors:** Ilaria Pagnoni, Elena Gobbi, Cristina Alaimo, Elena Campana, Roberta Rossi, Rosa Manenti, Michela Balconi, Maria Cotelli

**Affiliations:** ^1^Neuropsychology Unit, IRCCS Istituto Centro San Giovanni di Dio Fatebenefratelli, Brescia, Italy; ^2^Unit of Psychiatry, IRCCS Istituto Centro San Giovanni di Dio Fatebenefratelli, Brescia, Italy; ^3^International Research Center for Cognitive Applied Neuroscience (IrcCAN), Catholic University of the Sacred Heart, Milan, Italy; ^4^Research Unit in Affective and Social Neuroscience, Department of Psychology, Catholic University of the Sacred Heart, Milan, Italy

**Keywords:** theory of mind (ToM), executive functions (EFs), social cognition, major depressive disorder, depression, social cognition

## Abstract

Patients suffering from major depressive disorder (MDD) experience difficulties in multiple cognitive and affective abilities. A large body of literature has argued that MDD patients show impaired executive functions (EFs) and deficits in theory of mind (ToM), the ability to infer the mental states of others. However, the relationship between ToM and EFs has been poorly investigated. The aim of this review is to provide an overview of studies that evaluated the association between ToM and EFs in patients with MDD diagnosis. A literature review was conducted to identify all published studies in which ToM and EFs measures were administered to individuals with MDD and in which the relationship between these two domains was investigated. Eleven studies were included, and for each study, we discussed the findings related to ToM, EFs, and the nature of the link between these two aspects. Most of the studies reported that patients with MDD, compared with healthy controls, showed significant impairments in both ToM and EFs abilities. Moreover, this review indicates the presence of a significant association between these two domains in MDD patients, supporting the evidences that executive functioning is important to perform ToM tasks. Although the results that emerged are interesting, the relationship between ToM and EFs in MDD needs further investigation.

## Introduction

Major depressive disorder (MDD) is recognized as one of the most common mental illnesses and it is associated with significant impairments in psychosocial functioning and cognitive complaints (1, 2). According to the Diagnostic and Statistical Manual of Mental Disorders, 5th Edition, MDD is diagnosed when an individual experiences at least 5 out of the following nine symptoms during the same 2-week period with significant distress and difficulties in daily living. At least one of the symptoms between (1) depressed mood or (2) loss of interest must be present, in addition to (3) weight loss or gain; (4) insomnia or hypersomnia; (5) psychomotor agitation or retardation; (6) fatigue or loss of energy; (7) feeling worthless or excessive/inappropriate guilt; (8) decreased concentration; (9) thoughts of death/suicide (3, 4). Cognitive deficits are widely acknowledged as an important aspect of MDD that could contribute to the functional impairment (2). In this regard, a number of studies have suggested that MDD is frequently associated with impairment in attention, executive functions (EFs), and in different aspects of learning and memory (5). Furthermore, an important feature of depression is a reduction of social functioning, characterized by decreased social interaction and impairment in emotional information processing (6). A widely studied aspect of social functioning is the “theory of mind” (ToM) (7, 8). ToM, one of the key aspects of social cognition, is defined as the ability to attribute mental states, such as beliefs and intentions, to others in order to predict, describe, and explain their behavior on the basis of such mental states (7, 9, 10). ToM ability involves two major processes: the social-perceptual process (i.e., the ability to decode the mental states of others based on observable social information, such as facial expression, tone of voice, etc.) and the social-cognitive process (i.e., the ability to reason about mental states by integrating contextual information about a person, such as experiences and knowledge, involving higher-order functioning) (11). Understanding these mechanisms is extremely important for a full account of the role of cognitive processes in ToM. ToM is a composite function, which involves memory, attention, perceptual recognition, language, EFs, emotion processing and recognition, empathy. Each of these cognitive abilities contributes significantly to a series of processes such as social stimulus perception, processing, interpretation, and response, necessary in order to understand and predict the behavior of others (12). These processes, despite being ruled by different brain networks, generally work together to produce reliable and adequate judgments about the mental states of others and are considered essential for a successful interpersonal interaction (13).

The interest in social cognition in depression is justified by the relationship between deficits in these skills and the impact of the interpersonal difficulties on the patient’s life (14). Several authors have postulated that cognitive difficulties may negatively affect social cognition and pronounced cognitive impairment may be associated with the severity of depressive symptoms (2, 15). Some studies on MDD patients underline that the deficits in executive functioning could explain impaired social information analysis and reduced social functioning, hypothesizing the existence of an association between ToM and EFs, even though the exact nature of this relation remains at least partially unknown (6, 16–18). EFs play an important role in the control and plan of many cognitive processes and in the regulation of goal-directed behavior, influencing a variety of atypical behaviors and predicting functional and social outcomes (19–21). Inhibitory control or cognitive flexibility are necessary to understand what people feel or think (22).

Executive dysfunction is commonly reported in MDD and it is usually described as a consequence of structural and functional abnormalities of the fronto-subcortical networks, including the dorsolateral prefrontal cortex (DLPFC), the ventrolateral prefrontal cortex (VLPFC), and the anterior cingulate cortex (ACC) (23–26). Reduced levels of the main excitatory neurotransmitter (glutamate) have been hypothesized to be responsible for the hypoactivation of these brain areas, which are implicated in EFs (27, 28). Interestingly, several studies described that the involvement of this neural system is critical for social cognition too, supporting the evidence that executive functioning is necessary to perform cognitive ToM tasks (6, 29–33).

However, although ToM and EFs appear to share a common neurological basis, an in-depth analysis of the association between these two domains in MDD could help design more accurate interventions. In light of these premises, the purpose of this review is to investigate the relationship between ToM abilities and EFs in MDD patients.

## Review of the literature

### Search strategies and study selection criteria

**FIGURE 1:**
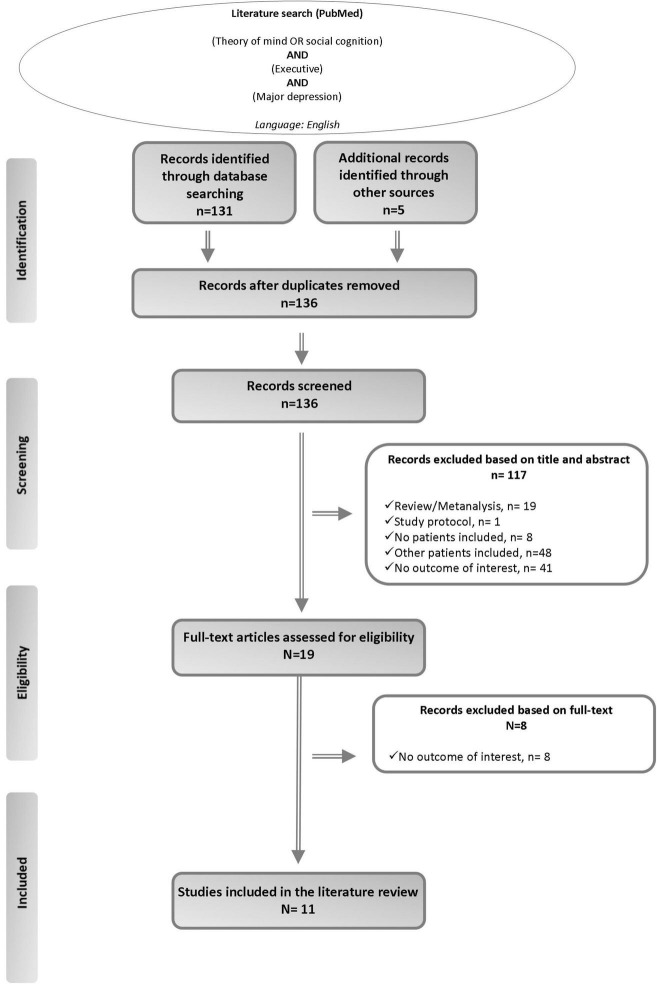
Summary of the literature search–PRISMA flow diagram (43).

A literature review was conducted to identify English-language studies in which both ToM and EFs were measured in individuals with MDD and in which the relationship between them was investigated. We searched the literature in the Medline (PubMed) database using the following terms: “(*theory of mind OR social cognition*) AND (*executive*) AND (*major depression*).” We screened all titles and abstracts and examined all pertinent research articles, including their references, to identify possible supplementary sources. From this study selection, animal studies, meta-analyses, reviews, study protocols, or letters were excluded. Thereafter, we proceeded to read the full texts of the remaining articles, applying the following inclusion criteria: (a) original research; (b) conducted on individuals with MDD; (c) providing an outcome measure of the relationship between ToM and EFs; and (d) published before 27th June 2022. We included studies that involved patients diagnosed with MDD regardless of severity (mild, moderate, severe), course (current, in partial or complete remission), and the presence/absence of psychotic features (Figure 1).

Of 19 full-text articles assessed for eligibility, 11 studies published between 2008 and 2019 fulfilled the criteria for inclusion in this literature review (Figure 1). Ten out of the eleven studies compared MDD patients to healthy subjects (6, 17, 18, 34–40), whereas only one study did not include a healthy control group (16) (Table 1).

**TABLE 1:** Studies that assessed the relationship between theory of mind (ToM) and executive functions (EFs) in major depressive disorder (MDD).

Study	Number of MDD subjects (Mean age in years, $\bar{x}$, and SD)	Number of control subjects (Mean age in years, $\bar{x}$ and SD)	Current/Remitted MDD	ToM measures and MDD difficulties	EFs measures and MDD difficulties	Relationship between ToM and EFs
Uekermann et al. (6)	27 MDD ($\bar{x}$ = 37.9 SD = 2.4)	27 HC ($\bar{x}$ = 37.6 SD = 1.9)	Current MDD (11/27 patients: one previous episode; 16/27 patients: first-episode)	MDD compared to HC: ↓ Humor Processing Computerized Task	MDD compared to HC: ↓ Letter-Number Sequencing subtest of Wechsler Memory Scale ↓ Trail Making Test (B) ↓ Stroop Test ↓ Regensburger Word Fluency Test	In MDD and HC groups, significant correlations between humor processing, mentalizing and EFs
Uekermann et al. (37)	29 MDD ($\bar{x}$ = 37.0 SD = 1.6)	29 HC ($\bar{x}$ = 39.1 SD = 2.4)	Current MDD (16/29 patients: one previous episode; 13/29 patients: first-episode)	MDD compared to HC: In Tübingen Affect Battery (a German adaptation of the Florida Affect Battery–Revised): ↓ Naming neutral semantic content ↓ Naming incongruent semantic prosody ↓ Matching of affective prosody to facial expression ↓ Matching of facial expression to affective prosody = Naming congruent semantic prosody	MDD compared to HC: ↓ Letter-Number Sequencing task of Wechsler Memory Scale ↓ Stroop Test ↓ Trail Making Test (A, B, and B–A)	In MDD and HC groups, significant correlations between affective prosody comprehension and EFs
Wang et al. (38)	23 Psychotic MDD ($\bar{x}$ = 26.8 SD = 4.4) 33 Non-psychotic MDD ($\bar{x}$ = 28.0 SD = 5.1)	53 HC ($\bar{x}$ = 25.7 SD = 3.6)	Current Psychotic MDD (first-episode) and Current Non-psychotic MDD (first-episode)	Psychotic MDD compared to HC and Non-psychotic MDD: ↓ Reading the Mind in the Eyes Test ↓ Faux Pas Task Non-psychotic MDD compared to HC: ↓ Reading the Mind in the Eyes Test ↓ Faux Pas Task	Psychotic MDD compared to HC and Non-psychotic MDD: ↓ Verbal Fluency test = Digit Span Test–Forward and Backward Non-psychotic MDD compared to HC: = Verbal Fluency test = Digit Span Test–Forward and Backward	In Psychotic and Non-psychotic MDD, significant correlations between ToM performance and EFs
Wolkenstein and colleauges (40)	24 MDD ($\bar{x}$ = 37.2 SD = 10.4)	20 HC ($\bar{x}$ = 35.7 SD = 11.2)	Current MDD	MDD compared to HC: Reading the Mind in the Eyes Test (RMET): = RMET–percent accuracy, total score ↑ RMET–percent accuracy, negative = RMET–percent accuracy, positive = RMET–percent accuracy, neutral Movie for the Assessment of Social Cognition (MASC): ↓ MASC–Correct ToM ↑ MASC–Less ToM = MASC–No ToM = MASC–Exceeding ToM	MDD compared to HC: = Trail Making test (TMT A and B) = Multiple Choice Word Fluency Test ↓ WCST (categories, errors, perseverations)	Positive correlations in MDD and HC groups: –between “Correct ToM” in MASC task and completed categories in the WCST; –between “Less ToM” in MASC task and the number of errors and perseveration in WCST Negative correlations in cMDD and HC groups: –between “Correct ToM” in MASC task and perseveration in the WCST; –between “Less ToM” in MASC task and completed categories in the WCST
Bertoux et al. (34)	19 MDD ($\bar{x}$ = 63.3 SD = 8.4)	30 HC ($\bar{x}$ = 66.2 SD = 9.9) 17 early bvFTD ($\bar{x}$ = 63.1 SD = 9.1) 20 moderate bvFTD ($\bar{x}$ = 66.7 SD = 8.3)	Not available	MDD compared to HC: Social Cognition and Emotional Assessment (SEA): ↓ SEA–composite score ↓ SEA–reversal learning and extinction test ↓ SEA–apathy scale from Starkstein = SEA–facial emotion recognition test = SEA–shortened version of the Faux Pas recognition test = SEA–behavioral control test = Mini–SEA MDD compared to patients in the early stage of bvFTD: Social Cognition and Emotional Assessment (SEA): ↑ SEA–composite score = SEA–reversal learning and extinction test ↑ SEA–apathy scale from Starkstein ↑ SEA–facial emotion recognition test ↑ SEA–shortened version of the Faux Pas recognition test = SEA–behavioral control test ↑ Mini–SEA MDD compared to patients in the moderate stage of bvFTD: Social Cognition and Emotional Assessment (SEA): ↑ SEA–composite score = SEA–reversal learning and extinction test ↑ SEA–apathy scale from Starkstein ↑ SEA–facial emotion recognition test ↑ SEA–shortened version of the Faux Pas recognition test ↑ SEA–behavioral control test ↑ Mini–SEA	MDD compared to HC: ↓ FAB MDD compared to patients in the early stage of bvFTD: = FAB = Verbal Fluency Test = WCST–categories = WCST–perseverations ↓WCST–errors MDD compared to patients in the moderate stage of bvFTD: ↑ FAB ↑ Verbal Fluency Test ↑ WCST–categories = WCST–perseverations = WCST–errors	In MDD group, significant correlations between SEA-reversal learning subtest and FAB
Szanto et al. (36)	24 MDD suicide attempters ($\bar{x}$ = 68.2 SD = 8.7) 38 non-suicidal MDD ($\bar{x}$ = 70.2 SD = 7.7)	28 HC ($\bar{x}$ = 69.6 SD = 6.3)	Current MDD suicide attempters and Current non-suicidal MDD	MDD suicide attempters compared to HC: ↓ Reading the Mind in the Eyes Test MDD non-suicidal attempters compared to HC: = Reading the Mind in the Eyes Test	MDD compared to HC: = Executive Interview (EXIT25)	In MDD suicide attempters, no significant correlations between social emotion recognition and executive performance
Ladegaard et al. (17)	44 MDD ($\bar{x}$ = 32.5 SD = 12.0)	44 HC ($\bar{x}$ = 32.9 SD = 12.0)	Current MDD (first-episode)	MDD compared to HC: ↓ FHA ↓ MAS-A = TASIT–Sincere subscale = TASIT–Simple subscale ↓ TASIT–Paradoxical sarcasm subscale	MDD compared to HC: ↓ CANTAB–Rapid Visual Information Processing subtest = CANTAB–Intra Extra Dimensional Shift subtest = CANTAB–One Touch Stockings subtest	Controlling for neurocognitive covariates did not change significant differences between the groups on the social cognitive tasks
Thoma et al. (18)	28 MDD ($\bar{x}$ = 43.4 SD = 11.7)	28 HC ($\bar{x}$ = 44.3 SD = 12.0)	Current MDD (15/28 patients: at least two previous episodes; 13/28 patients: first-episode)	MDD compared to HC: Mentalistic Interpretation Task: = Control questions ↓ Quality of the interpretation of sarcastic items = Quality of the interpretation of physical items = Quality of the interpretation of mentalistic items = Selection of best interpretation out of a range of alternatives for all items –Social Problem Resolution Task: = Control questions ↓ Quality of solutions generated for social problems ↓ Social and practical solutions = Social not practical solutions = Practical not social solutions = Neither social nor practical solutions –Social Problem Solving Fluency Task: = Control questions = Detection of awkward elements = Subjective judgment of the awkwardness of situations ↓ Quality of solutions generated for awkward social situations ↓ Social and practical solutions ↓ Social not practical solutions = Practical not social solutions = Neither social nor practical solutions = Selection of best alternatives	MDD compared to HC: = GoNogo subtest from the Test Battery of Attentional Functions = Working Memory subtest from the Test Battery of Attentional Functions No results are reported for Trail Making Test (A and B)	In MDD group, significant correlations between generation scores and TMT
Wang et al. (39)	35 MDD ($\bar{x}$ = 29.4 SD = 7.9)	35 HC ($\bar{x}$ = 27.3 SD = 6.7) 35 SCZ ($\bar{x}$ = 29.1 SD = 5.8) 35 BD ($\bar{x}$ = 31.1 SD = 6.8)	Not available	MDD compared to HC Yoni Task-First-order conditions: = Cognitive condition = Affective condition Yoni Task-Second-order conditions: ↓ cognitive condition ↓ affective condition	MDD compared to HC: ↓ Letter–Number Span Test MDD compared to SCZ and BD: ↑ Letter–Number Span Test	In MDD, SCZ, BD significant correlations between second-order affective ToM performance, depressive and psychotic symptoms and EFs
Förster et al. (35)	118 MDD ($\bar{x}$ = 20.6 SD = 3.8)	61 HC ($\bar{x}$ = 19.1 SD = 2.4)	48 Remitted MDD and 70 Current MDD	Remitted and Current MDD compared to HC: = ACS for WAIS-IV and WMS-IV–Affect naming = ACS for WAIS-IV and WMS-IV–Prosody face matching = ACS for WAIS-IV and WMS-IV–Prosody-pair matching = ACS for WAIS-IV and WMS-IV–total score	Remitted and Current MDD compared to HC: = CATS–Card Sort Task = CATS–Tower of London = CATS–N-back-task = CATS–Victoria Stroop Test	In Current MDD, social cognition total score and affect score were significantly associated with EFs In Remitted MDD, no significant correlations between social cognition and EFs
Knight and Baune (16)	111 MDD ($\bar{x}$ = 35.0 SD = 16.4)	None	69 Remitted MDD and 42 Current MDD (For all patients: mean number of previous episodes was 1.4)	ACS for WAIS-IV and WMS-IV–Affect naming ACS for WAIS-IV and WMS-IV–Prosody face matching ACS for WAIS-IV and WMS-IV–Prosody-pair matching ACS for WAIS-IV and WMS-IV–total score	CATS–Wisconsin Card Sorting Test CATS–Tower of London PEBL–Stroop Test PEBL–Tower of London PEBL–Wisconsin Card Sorting Test	Indirect relationship between theory of mind abilities and psychosocial dysfunction, as mediated by executive functioning

↑, greater performance; ↓, worse performance; =, equal performance; $\bar{x}$, mean; ACS, advanced clinical solutions; BD, bipolar disorder; bvFTD, behavioral variant of Fronto-temporal dementia; CANTAB, cambridge neuropsychological test automated battery; CATS, colorado assessment test; FAB, frontal assessment battery; FHA, Frith-Happé animations; HC, healthy controls; MAS-A, metacognition assessment scale-abbreviated; MDD, major depressive disorder; PEBL, psychological experiment building language; SCZ, schizophrenia; SD, standard deviation; TASIT, the awareness of social inference test; ToM, theory of mind; WAIS-IV, wechsler adult intelligence scale-fourth edition; WCST, wisconsin card sorting test; WMS-IV, wechsler memory scale-fourth edition.

The selected studies considered a total of 553 MDD patients and 355 healthy controls. Specifically, the MDD patients did not show other Axis I disorders and had not history of personality disorders; only one study involved patients with MDD with comorbid substance use disorders (36) and likewise, only one study described Axis II disorders in MDD patients (17). Moreover, seven studies considered patients with current diagnosis of MDD (6, 17, 18, 36–38, 40), two studies described MDD patients with current or previous episodes (16, 35) and two studies did not specify the stage of MDD (34, 39).

### Characteristics of included studies

Below we detailed the evidences from the literature about the relationship between ToM and EFs in MDD patients (Table 1).

Uekermann et al. (6), investigated the link between humor processing, mentalizing, and EFs in current MDD patients. Results showed that patients with MDD had significantly more difficulty than healthy controls in correctly answering questions that relate to the perspective of other people, suggesting the presence of impairments in cognitive and affective components of humor processing and in mentalizing ability. Moreover, there was a significant difference between the two groups on tasks used for assessing EFs, observing significantly lower performance in patients with MDD. Finally, significant correlations between humor processing, mentalizing and EFs in all participants were found. In a subsequent study, the authors assessed the potential contribution of executive deficits to impairments of affective prosody perception in current MDD. Significant differences were observed between MDD patients and healthy controls, indicating a significantly poorer performance of MDD patients in most tasks that assessed the perception of affective prosody and in the measures of EFs. Moreover, significant correlations between these two components were found both in control group and in MDD group, suggesting that the affective comprehension deficits in MDD group may be influenced by EFs (37). Wang et al. (38) investigated whether psychotic symptoms in current MDD were associated with lowest performance on ToM tasks. This study reported that psychotic MDD patients performed significantly worse than non-psychotic current MDD patients and healthy controls on tasks involving ToM social-perceptual and ToM social-cognitive components, and that non-psychotic MDD patients had significantly lower performance than normal controls in these tasks. Conversely, psychotic MDD patients showed more significant difficulties than healthy subjects and non-psychotic MDD patients in a task that assessed EFs, while no significant differences between non-psychotic MDD patients and healthy controls were found. Interestingly, the researchers reported significant correlations between ToM performance and EFs in all MDD patients. Wolkenstein and colleauges (40) investigated in patients with current episode of MDD, two aspects that characterize ToM ability, respectively the process of decoding mental states from observable social information and reasoning about mental states. Compared to healthy subjects, their findings indicated that MDD patients did not show a significant decreased ability to decode mental states when the total ToM task score was considered. However, it is important to note that in the study, MDD patients were significantly more accurate than healthy controls for negative stimuli, while no significant differences between the groups were found for positive and neutral stimuli. Regarding the reasoning abilities, MDD patients compared to healthy subjects, exhibited significant difficulties in the selection of correct mental state, answering more often in an insufficient manner. In addition, the authors found that MDD patients significantly performed worse than the healthy group in an EFs task. Interestingly, both positive and negative correlations were found between the ToM-reasoning ability measures and the executive task in all groups. In a study aimed to evaluate the sensitivity of social and emotional cognition measures (Social Cognition and Emotional Assessment–SEA and Mini-SEA) for differentiating behavioral variant of Fronto-temporal dementia (bvFTD) from MDD patients, Bertoux and coworkers (34) showed that the performance of the MDD group in some SEA subtests was significantly lower than healthy controls, but their performance was significantly better than patients in the early and moderate stages of bvFTD. Regarding the EFs assessment, the authors observed that MDD patients performed significantly worse than healthy controls and subjects in the early stage of bvFTD subjects, but performed significantly better than patients in the moderate stage of bvFTD patients. Furthermore, significant correlations were found between a subtest of SEA and an EFs measure in MDD patients, suggesting a relationship between these two domains. Szanto et al. (36) conducted a study on older current MDD patients with or without suicide attempts, in order to examine whether emotion regulation and social functioning were associated with attempted suicide. Focusing on social emotion recognition, the researchers reported that MDD suicide attempters provided significantly more errors in a ToM task, compared to healthy controls, whereas non-suicidal MDD participants showed intermediate performance that did not significantly differ from MDD suicide attempters subjects and healthy controls. Differently, no significant statistical differences were found between the three groups in a measure of executive functioning. Furthermore, the authors carried out a correlation analysis only for MDD suicide attempters, reporting significant correlations between ToM and global cognitive performance, but not with executive measures. Differently, Ladegaard and colleagues (17) investigating higher-order social cognition in current MDD, pointed out that MDD patients significantly performed worse than healthy controls in a mental state attribution scale, in the social perception task, and in a subscale of metacognition measure. Furthermore, MDD patients exhibited a significantly poorer performance compared to healthy participants in a sustained attention task. Investigating the possible explanatory variables for socio-cognitive deficits, the authors reported that neurocognitive performance did not change the significant differences between the groups on higher-order social cognition tasks. In a study by Thoma et al. (18), different aspects of problem solving were assessed in a group of current MDD patients. The results demonstrated that MDD patients showed significantly more difficulties than healthy controls in the interpretation quality of other people’s sarcastic utterances as well as generated significantly fewer socially sensitive and practical solutions in response to the social problems. Otherwise, no significant group differences were found in the performances on the attentional tasks. Moreover, in order to investigate the possible correlations between social problem solving measures and EFs tasks in MDD group, the researchers generated two composite scores, generation and judgment, based on key measures in each of the three social cognition tasks used. Respectively, the generation score measured the ability to freely generate solutions to interpersonal problems, while the judgment score referred to the identification of the best solutions among less optimal alternatives. Findings showed significant correlations between generation score and cognitive flexibility, suggesting a role of EFs in social problem solving. Furthermore, a recent study by Wang et al. (39) evaluated cognitive and affective components of ToM at the first- and second-order level in three groups of patients with a diagnosis of schizophrenia, bipolar disorder, and MDD. This study showed that MDD performed significantly worse than healthy subjects only in the second-order cognitive and affective conditions. In addition, the assessment of EFs highlighted significant differences between the groups: MDD patients showed more difficulty than healthy controls, but less than schizophrenic or bipolar patients. Moreover, the existence of significant correlations between second-order affective ToM performance, depressive and psychotic symptoms, and EFs were described in all patient groups, suggesting that impairment in second-order affective ToM was associated with higher levels of depression and psychotic symptoms, and with poorer EFs. In their study, Förster et al. (35) investigated the relationship between social cognitive performance and EFs in patients with remitted and current MDD, showing that both groups of MDD patients, compared with the healthy controls group, did not significantly differ in any domain of social cognitive performance and of EFs. However, investigating the link between these two domains, the authors found that only for patients, ToM and affective perception were significantly associated with EFs. Finally, Knight and Baune (16) conducted a mediation analysis in order to understand the relationship between social cognition and psychosocial dysfunction in patients with a current or previous episode of MDD. They found an indirect relationship between ToM and total psychosocial dysfunction, as mediated by executive functioning, indicating that meaning interpretation is related to EFs, which in turn is related to perceived cognitive performance.

#### Summary

Ten of eleven studies described the performance of healthy subjects and patients with MDD on measures of ToM and EFs (6, 17, 18, 34–40), while only one study did not include a healthy control group (16). Regarding studies that compared MDD patients with healthy controls, nine out of ten studies reported a significantly worse performance of MDD patients on at least one component of ToM (6, 17, 18, 34, 36–40), whereas, seven out of ten studies showed that MDD individuals had significantly poorer performance on tasks that assessed executive functioning (6, 17, 34, 37–40). Finally, nine out of eleven studies found a significant relationship between ToM and EFs in MDD individuals (6, 16, 18, 34, 35, 37–40). However, the two studies that did not find a significant association between these two domains showed interesting results (17, 36): a study described significant correlations between ToM impairment and global cognitive decline in MDD suicide attempters (36), while the other one reported that controlling for neurocognitive measures as covariates, no significant differences were found on social cognition tasks in both MDD patients and healthy subjects, suggesting that social cognition is relatively independent from non-social domain (17).

Overall, with respect to the primary aim of this review, we found support for our hypothesis of association between ToM and EFs in MDD patients. In particular, a study has shown that MDD patients with better cognitive flexibility generate more solutions for solving complex social situations (18). Moreover, Förster et al. (35) reported that overall social cognition abilities, including ToM, were positively related to cognitive flexibility only in patients with current MDD diagnosis and not in patients with remitted MDD diagnosis. Wolkenstein and colleagues (40) found a significant correlation between a specific component of ToM, the ability to reason about mental states, and an EFs measure in MDD patients. Interestingly, a recent study suggested that the relationship between social cognitive deficits and psychosocial dysfunction was not mechanistically explained by mood symptoms, but seems to be mediated by EFs (16).

## Discussion

The present work aimed to provide a comprehensive review of the literature that investigate the relationship between ToM and EFs in patients with diagnosis of MDD. Most of the studies included in the present review reported that MDD patients, compared to healthy controls, showed an impairment in both ToM and EFs measures (6, 17, 34, 37–40) and highlighted an association between these two domains (6, 16, 18, 34, 35, 37–40). Some preliminary data justify the goals of this literature review, since previous studies on MDD patients have shown that attention and EFs are the cognitive domains most associated with impaired social functioning (2). A determinant aspect of social functioning is ToM, but the findings about the association between ToM and EFs in MDD patients are still heterogeneous and in some cases they are even mixed (41, 42). In this regard, in a recent meta-analysis aimed to investigate the variables could be able to influence the performance on ToM measures, the authors reported only a trend-level relationship between ToM impairment and executive dysfunction in individuals with MDD. Most of the reviewed studies recruited patients with current MDD, and interestingly one study compared patients with remitted and current MDD, showing a relationship between EFs and ToM selectively in patients with a current diagnosis of major depression (35). The evidence of a crucial role of EFs in ToM tasks might be relevant in clinical practice. In particular, following the results of our literature review, these abilities should be deeply investigated in order to improve both diagnostic pathway and the selection of the better individualized treatment strategy in MDD patients.

Although the results of this review seem interesting, some limitations which do not allow for definitive conclusions must be considered. First, the limited number of studies found in the literature allows us to provide only preliminary evidences regarding the nature of the link between ToM and EFs in patients with MDD. Second, the heterogeneity of patients with respect to the severity and course of the disorder, pharmacological treatment and the presence of psychotic features or suicidal ideation may have influenced scores on cognitive tasks and should be considered in the interpretation of the results. Finally, many different ToM and EFs measures were used across studies, thereby complicating the identification of a firm conclusion. To sum up, the relationship between ToM and EFs in patients with MDD is an underdeveloped topic in the literature, but represents an highly relevant issue for both clinical and research purposes. Clearer findings on the nature of this association in this category of patients would play a key role in rehabilitation. In the future, it would be interesting to investigate whether the enhancement of EFs, also using neuromodulation techniques such as transcranial direct current stimulation (tDCS) combined with cognitive training, would bring benefits in terms of social cognition in patients with MDD.

Future studies should better explain the direction of the relationship between ToM and EFs in this patient population, also considering the different stage of MDD.

## Author contributions

IP, EG, RM, MB, and MC: conception and methodology. IP, EG, CA, RM, MB, and MC: writing – original draft preparation. IP, EG, CA, EC, RR, RM, MB, and MC: writing – review and editing. All authors have read and agreed to the published version of the manuscript.
